# Prevalence of depression among rural older adults in Asia: a meta-analysis of observational studies

**DOI:** 10.3389/fpubh.2026.1902088

**Published:** 2026-07-08

**Authors:** Ting Li, Jiaxin He, Yu Mao, Huixiang Li

**Affiliations:** 1 West China Center of Excellence for Pancreatitis, Institute of Integrated Traditional Chinese and Western Medicine, West China Hospital, Sichuan University/West China School of Nursing, Sichuan University, Chengdu, China; 2 Department of Anesthesiology, Public Health Clinical Center of Chengdu, Chengdu, China; 3 Division of Surgery, Institute of Integrated Traditional Chinese and Western Medicine, West China Hospital, Sichuan University/West China School of Nursing, Sichuan University, Chengdu, China

**Keywords:** Asia, depression, meta-analysis, older adults, prevalence, rural areas

## Abstract

**Background:**

Despite the prevalence of depression among rural older adults in Asia having been widely investigated, existing estimates remain highly inconsistent. This meta-analysis of observational studies aimed to quantify the pooled prevalence of depression and determine potential moderating factors among rural older adults in Asia.

**Methods:**

A systematic literature search was performed in PubMed, Web of Science, Scopus, Embase, Cochrane Library, CINAHL, and PsycINFO from inception to May 2026. Pooled prevalence estimates were calculated using a random-effects model, with both 95% confidence intervals and 95% prediction intervals reported for each estimate. Heterogeneity was assessed using the I^2^ statistic, and subgroup analyses together with meta-regression were conducted to identify potential sources of heterogeneity.

**Results:**

A total of 68 studies comprising 58,577 rural older adults in Asia were included, yielding a pooled prevalence of depression of 34.6% (95% CI: 29.9–39.6%, 95% PI: 3.9–75.9%, *I*^2^ = 99.3%). The pooled prevalence was significantly associated with mean age, proportion of married participants, economic status, and geographic region (all *p* < 0.05).

**Conclusions:**

Our findings indicate that depression is common among rural older adults in Asia. The pooled prevalence estimate exhibited substantial statistical heterogeneity and a wide prediction interval, only partly accounted for by covariates such as mean age, proportion of married participants, economic status, and geographic region. Therefore, caution is advised when interpreting this estimate in clinical practice. Furthermore, the substantial mental health burden in this population highlights the urgent need for routine screening and tailored interventions to reduce depression. **Systematic review registration:** CRD420261411159.

## 1 Introduction

The global population is undergoing rapid aging, with Asia at the forefront of this demographic transition (1). Asia accounts for more than half of the world's population aged 60 years and older, and this proportion is projected to continue rising in the coming decades (2). This profound demographic transition poses considerable challenges to the social, economic, and healthcare systems in Asian countries (3). Depression, defined as a mental disorder characterized by persistent sadness, loss of interest, feelings of guilt or low self-worth, disturbed sleep or appetite, and poor concentration, is one of the most common mental health conditions among older adults (4, 5). Moreover, late-life depression not only impairs quality of life and physical functioning but also increases the risk of suicide, cognitive decline, and mortality, imposing a substantial burden on individuals, families, and healthcare systems (6, 7).

Rural older adults represent a particularly vulnerable subgroup that may face a higher risk of depression compared with their urban counterparts (8). Rural areas are generally characterized by limited access to mental health services, lower socioeconomic status, higher prevalence of chronic diseases, weaker social support networks, and greater social isolation (9, 10). These unique risk factors distinguish rural older adults from the general elderly population and necessitate a separate examination of their depression burden. Furthermore, Asian settings require distinct consideration from Western or global contexts. In many Asian countries, the out-migration of younger generations to urban areas for employment has resulted in a growing number of empty-nest older adults living alone in rural villages, further exacerbating feelings of loneliness and depression (11). In addition, mental health literacy is low in rural communities, and stigma associated with mental illness remains strong, discouraging help-seeking behavior and leading to underdiagnosis and undertreatment of depression (12). Therefore, accurately estimating the prevalence of depression among rural older adults in Asia and identifying its moderating factors are of great public health significance for developing targeted prevention strategies and reducing the disease burden in this vulnerable population.

Over the past two decades, numerous observational studies have reported the prevalence of depression among rural older adults across various Asian countries. Many of these studies have employed age-specific screening instruments, such as the 30-item and 15-item versions of the Geriatric Depression Scale, which are designed to capture depression symptoms specifically in later life and demonstrate superior sensitivity and specificity for detecting depression in older populations compared to other generic depression measures (13, 14). However, the reported estimates vary widely, ranging from 1.7% to 79.4% (13–17). This considerable heterogeneity may be attributed to differences in geographic regions, economic status, screening instruments, and sample characteristics. To date, no comprehensive meta-analysis has systematically synthesized the available evidence to provide a robust pooled estimate of depression prevalence specifically among rural older adults in Asia, nor has the extent to which these covariates explain the observed heterogeneity been quantitatively examined. Moreover, while previous meta-analyses have quantified the global prevalence of depression in older adults (18, 19), none have specifically targeted rural older populations in Asia. This leaves a critical gap in evidence-based policymaking for mental health services in these underserved communities. The present meta-analysis therefore provides the first pooled prevalence estimate for this population and quantitatively examines key moderators to inform targeted interventions. Therefore, this meta-analysis of observational studies aimed to determine the pooled prevalence of depression and identify potential moderating factors among rural older adults in Asia.

## 2 Methods

We conducted this meta-analysis in accordance with the Preferred Reporting Items for Systematic Reviews and Meta-Analyses (PRISMA) guidelines (20), and the protocol was registered with PROSPERO (CRD420261411159).

### 2.1 Search strategy

A thorough search of PubMed, Web of Science, Scopus, Embase, Cochrane Library, CINAHL, and PsycINFO was performed from database inception to May 2026 (Supplementary Table S1). The search syntax did not apply language filters, but screening was restricted to English-only studies owing to the absence of multilingual translation resources within the research team. Employing Boolean logic, the search strategy integrated MeSH terms and free keywords, such as rural population, geriatrics, elderly, older adults, depression, and prevalence. Additional eligible studies were identified by manually reviewing the reference lists of all included studies.

### 2.2 Eligibility criteria

Studies were eligible for inclusion if they met the following criteria: (1) participants consisted of rural-dwelling older adults (≥ 60 years) in Asia; (2) a cross-sectional or longitudinal design was employed; (3) depression was assessed using the GDS-30 or GDS-15; (4) prevalence of depression was reported, or sufficient raw data were provided to allow its calculation. Conference abstracts, study protocols, case reports, reviews, duplicate publications, studies not published in English, as well as gray literature including dissertations and non-peer-reviewed papers, were excluded.

### 2.3 Study selection and data extraction

Data were independently extracted by two reviewers, with disagreements resolved through consultation with a third reviewer. The extracted information included first author, publication year, country, study design, sample size, mean age, proportions of females and married participants, screening tool, and depression prevalence.

### 2.4 Risk of bias assessment

Two reviewers independently assessed the risk of bias of each included study using the Joanna Briggs Institute (JBI) critical appraisal checklist for prevalence studies (21), which covers nine domains. Each item was rated as “yes” (low risk), “no” (high risk), or “unclearor not applicable.” Studies were classified as low (≥ 70% “yes”), moderate (50%-69%), or high ( ≤ 49%) risk of bias. Any disagreements were resolved by consulting a third reviewer until consensus.

### 2.5 Data analysis

Data analyses were conducted with Stata 19.0. Prior to meta-analysis, a Freeman-Tukey double arcsine transformation was applied to normalize the distribution and stabilize the variance of the prevalence estimates. Heterogeneity was assessed using the I^2^statistic, with ≥ 50% indicating substantial heterogeneity. A random-effects model employing restricted maximum likelihood (REML) estimation was then applied to estimate the pooled prevalence and its 95% confidence interval (CI). In addition, the 95% prediction interval (PI) was calculated to illustrate the expected range of true prevalence estimates in future studies. To identify potential sources of heterogeneity, subgroup analyses were conducted by economic status, geographic region, and screening tool, alongside univariate meta-regression assessing the relationship between the pooled prevalence and covariates (publication year, sample size, mean age, sampling strategy, proportion of females, proportion of married participants, economic status, geographic region, screening tool, and risk of bias). Economic status was classified according to the World Bank classification system for the fiscal year 2024, based on gross national income per capita: high-income, upper-middle-income, and low-middle-income. We evaluated publication bias using funnel plots and Egger's test, with the trim and fill method applied to correct for potential bias when indicated. The robustness of the pooled estimates was further examined through a series of sensitivity analyses, including leave-one-out analysis, exclusion of studies judged to be at moderate risk of bias, and exclusion of studies with missing covariate data (mean age, proportion of females, and proportion of married participants). Statistical significance was set at two-sided *P* < 0.05.

## 3 Results

### 3.1 Literature selection and characteristics

After an initial database search, a total of 754 records were identified. Following the removal of 353 duplicate entries, 401 records remained for title and abstract screening. Of these, 308 records were excluded, leaving 93 full-text articles for eligibility assessment. During the full-text review, 25 articles were excluded due to reasons such as insufficient data, non-English publication, or inappropriate study design. Ultimately, 68 studies met all the inclusion criteria and were incorporated into the meta-analysis.

**Figure 1:**
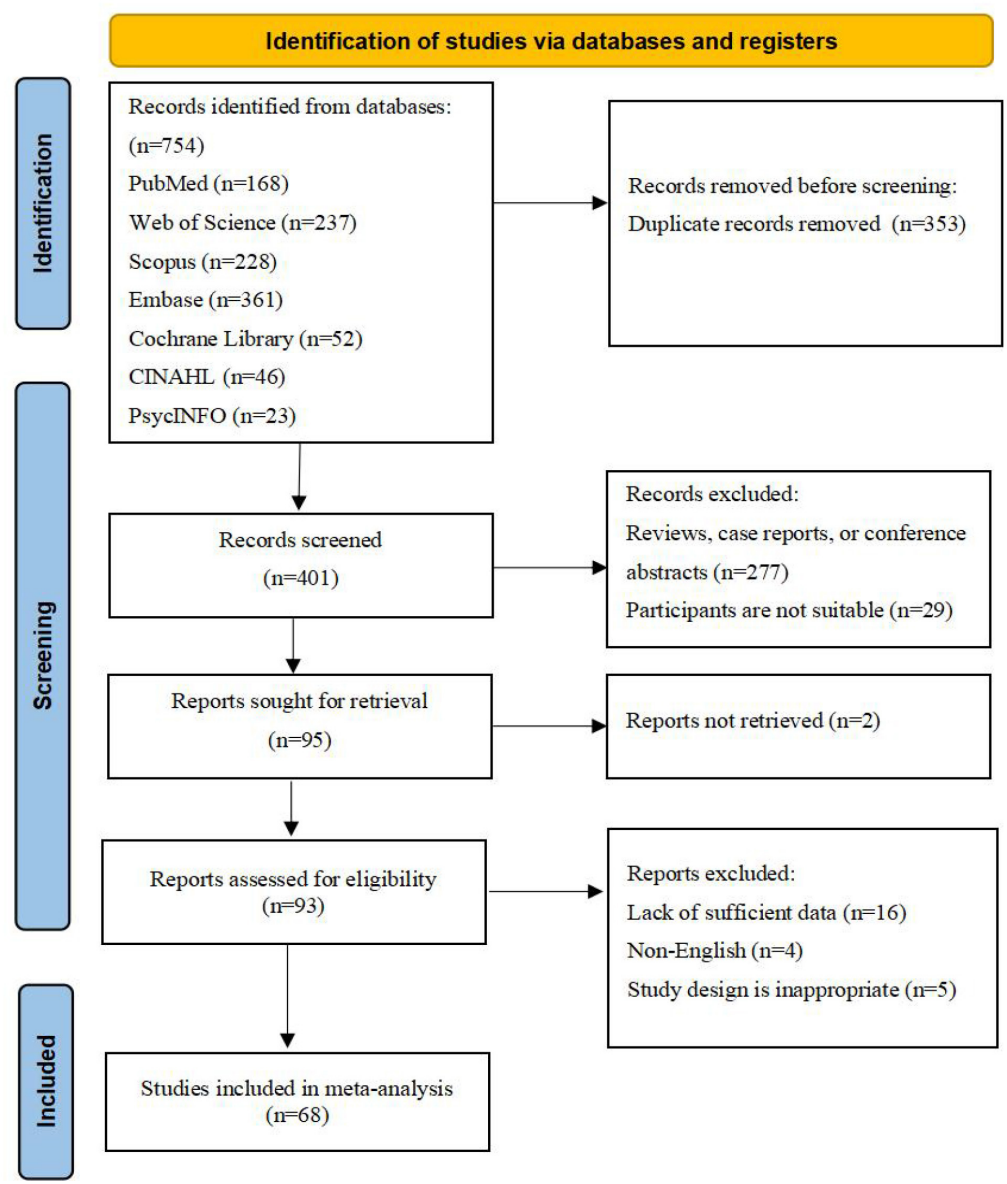
Flow diagram of study selection.

The study selection process is provided in Figure 1. Although longitudinal studies were also eligible, none met the inclusion criteria because they did not report baseline prevalence separately. Therefore, a total of 68 cross-sectional studies involving 58,577 rural older adults in Asia were included. These studies were conducted across 14 countries, including China, India, Japan, South Korea, Bangladesh, Nepal, Vietnam, Saudi Arabia, Pakistan, Turkey, Thailand, Malaysia, Iran, and Indonesia. Sample sizes ranged from 84 to 5,863 participants. Depression was assessed primarily using the Geriatric Depression Scale (GDS), including the GDS-15 and GDS-30 versions. The reported prevalence of depression varied substantially across studies, ranging from 1.7% to 79.4%. Detailed characteristics of each included study are presented in Table 1.

**Table 1:** Characteristics of included studies.

Reference	Country	Study design	Sample size	Mean age (years)	Married (%)	Females (%)	Screening tool	Prevalence (%)
Abe et al. (13)	Japan	Cross–sectional	1,041	76.7	NA	47.3	GDS−15	24.7
Al–Shammari et al. (14)	Saudi Arabia	Cross–sectional	2,525	NA	NA	NA	GDS−30	44.0
Akila et al. (15)	India	Cross–sectional	255	71.4	66.3	63.5	GDS−15	32.6
Ali et al. (16)	Pakistan	Cross–sectional	214	NA	NA	NA	GDS−15	79.4
Antony et al. (22)	India	Cross–sectional	479	NA	66.2	55.8	GDS−15	44.4
Behera et al. (23)	India	Cross–sectional	395	69.2	61.3	56.5	GDS−30	11.4
Buvneshkumar et al. (24)	India	Cross–sectional	690	68.1	56.7	52.6	GDS−30	35.5
Çakmur H. (25)	Turkey	Cross–sectional	168	72.7	NA	53.6	GDS−15	10.7
Charoensakulchai et al. (26)	Thailand	Cross–sectional	416	69.3	78.1	54.1	GDS−30	18.5
Chiu et al. (27)	China	Cross–sectional	327	72.4	64.2	50.8	GDS−15	12.8
Chuang et al. (28)	China	Cross–sectional	122	72.6	77.0	58.7	GDS−15	8.2
Dasgupta et al. (29)	Bangladesh	Cross–sectional	85	NA	63.5	63.5	GDS−15	58.8
Devaraj et al. (30)	India	Cross–sectional	114	69.3	61.4	56.1	GDS−15	9.6
Disu et al. (31)	Bangladesh	Cross–sectional	84	NA	NA	NA	GDS−15	44.0
Do et al. (32)	Vietnam	Cross–sectional	495	NA	65.1	68.7	GDS−15	28.7
Dong et al. (33)	China	Cross–sectional	736	NA	65.4	55.2	GDS−30	26.2
Fan et al. (34)	China	Cross–sectional	439	NA	NA	NA	GDS−30	13.7
Fukunaga et al. (35)	Japan	Cross–sectional	964	NA	52.7	61.9	GDS−15	20.6
Gao et al. (36)	China	Cross–sectional	1,737	74.3	58.5	53.1	GDS−30	30.8
Gao et al. (37)	China	Cross–sectional	501	69.7	NA	52.1	GDS−30	51.5
Gong et al. (38)	China	Cross–sectional	3,182	70.7	42.8	66.8	GDS−15	41.8
Goswami et al. (39)	India	Cross–sectional	290	NA	71	55.5	GDS−30	41.7
Guo and Shi. (40)	China	Cross–sectional	291	70.4	69.4	57.7	GDS−15	12.4
Hairi et al. (41)	Malaysia	Cross–sectional	765	NA	63.3	62.6	GDS−15	22.7
He et al. (42)	China	Cross–sectional	509	NA	NA	30.1	GDS−30	36.9
Hossain et al. (43)	Bangladesh	Cross–sectional	472	62.9	58.2	52.8	GDS−15	54.6
Hu et al. (44)	China	Cross–sectional	695	72.6	NA	61.9	GDS−15	25.0
Kaphle et al. (45)	Nepal	Cross–sectional	397	70	76.8	36.3	GDS−15	29.0
Kim et al. (46)	Korea	Cross–sectional	649	NA	NA	NA	GDS−30	33.3
Kumar et al. (47)	India	Cross–sectional	134	66	NA	NA	GDS−15	72.0
Kumar et al. (48)	India	Cross–sectional	121	NA	94.8	32.2	GDS−15	38.0
Kumari et al. (49)	India	Cross–sectional	425	67.5	76.0	56.5	GDS−15	29.2
Laksham et al. (50)	India	Cross–sectional	359	67.4	61.5	57.4	GDS−15	68.5
Liu et al. (51)	China	Cross–sectional	1,313	73.9	NA	55.5	GDS−15	12.9
Mahanta et al. (52)	India	Cross–sectional	180	NA	NA	NA	GDS−30	26.1
Malla et al. (53)	Nepal	Cross–sectional	387	NA	51.2	53.0	GDS−15	72.9
Manandhar et al. (54)	Nepal	Cross–sectional	222	NA	NA	NA	GDS−15	63.5
Maraqa et al. (55)	Palestine	Cross–sectional	208	NA	NA	NA	GDS−15	52.9
N S. (56)	India	Cross–sectional	400	NA	NA	50.0	GDS−15	47.0
Nagoor et al. (57)	India	Cross–sectional	415	67.2	68.9	52.1	GDS−15	27.7
Nahcivan et al. (58)	Turkey	Cross–sectional	132	64.8	71.2	54.5	GDS−30	50.0
Nair et al. (59)	India	Cross–sectional	366	NA	51.4	53.6	GDS−15	61.2
Naveen et al. (60)	India	Cross–sectional	411	NA	61.8	47.9	GDS−15	19.7
Nguyen et al. (61)	Vietnam	Cross–sectional	2,509	NA	NA	NA	GDS−15	24.3
Papadopoulos et al. (62)	Iran	Cross–sectional	608	NA	21.7	54.2	GDS−15	38.8
Park et al. (63)	Korea	Cross–sectional	3,041	NA	77.6	57.3	GDS−15	18.9
Patel et al. (64)	India	Cross–sectional	166	NA	NA	NA	GDS−15	75.3
Patil et al. (65)	India	Cross–sectional	388	68.1	67.3	66	GDS−15	34.0
Pilania et al. (66)	India	Cross–sectional	500	68.5	64.8	53.8	GDS−30	14.4
Rahman et al. (67)	Bangladesh	Cross–sectional	223	NA	NA	NA	GDS−15	48.4
Rai et al. (68)	India	Cross–sectional	4,493	58.9	NA	52.4	GDS−30	14.5
Reddy et al. (69)	India	Cross–sectional	800	NA	49.3	50	GDS−15	47.0
Ren et al. (70)	China	Cross–sectional	4,845	70.3	NA	57.3	GDS−15	10.4
Rong et al. (71)	China	Cross–sectional	3,349	71.2	NA	50.8	GDS−30	52.9
Roy et al. (72)	India	Cross–sectional	292	NA	53.1	70.2	GDS−15	64.1
Ruan et al. (73)	China	Cross–sectional	327	72.6	61.5	59	GDS−15	24.5
Sahni et al. (74)	India	Cross–sectional	162	NA	90.7	44.4	GDS−15	40.7
Sengupta et al. (75)	India	Cross–sectional	1,248	NA	NA	NA	GDS−15	7.3
Seo et al. (76)	Korea	Cross–sectional	881	70.3	63.6	67.0	GDS−15	25.3
Sinha et al. (77)	India	Cross–sectional	103	NA	61.2	43.7	GDS−15	42.7
Sirohi et al. (78)	India	Cross–sectional	456	69.4	NA	56.1	GDS−30	27.8
Soenarti et al. (17)	Indonesia	Cross–sectional	400	NA	NA	70.3	GDS−15	1.7
Vafaei et al. (79)	Iran	Cross–sectional	370	70.6	NA	64.9	GDS−15	50.3
Wada et al. (80)	Japan	Cross–sectional	5,863	NA	NA	53.4	GDS−15	33.5
Wu et al. (81)	China	Cross–sectional	2,000	68.8	63.4	59.9	GDS−15	28.9
Xie et al. (82)	China	Cross–sectional	415	NA	55.7	44.3	GDS−30	74.4
Yadav et al. (83)	Nepal	Cross–sectional	794	69.9	53.5	49.6	GDS−15	55.8
You et al. (84)	China	Cross–sectional	234	73.3	NA	100	GDS−15	44.0

NA, Not Available; GDS-30:Geriatric Depression Scale-30; GDS-15:Geriatric Depression Scale-15.

### 3.2 Risk of bias assessment results

Based on the JBI criteria, 52 (76.5%) studies were rated as having a low risk of bias, while the remaining 16 studies (23.5%) were rated as having a moderate risk of bias. Regarding individual quality domains, the sample frame was appropriate in 65 (95.6%) studies, and the sampling method was appropriate in 57 studies (83.8%). An adequate sample size was reported in 36 (52.9%) studies, whereas it was unclear in 32 (47.1%) studies. All 68 studies provided a detailed description of study subjects and setting, achieved sufficient coverage of the identified sample for data analysis, used valid methods for condition identification, measured the condition in a standard and reliable manner for all participants, and conducted appropriate statistical analysis. The response rate was adequate in 52 (76.5%) studies, inadequate in 4 (5.9%) studies, and unclear in the remaining 12 (17.6%) studies.

### 3.3 Pooled prevalence of depression among rural older adults in Asia

**Figure 2:**
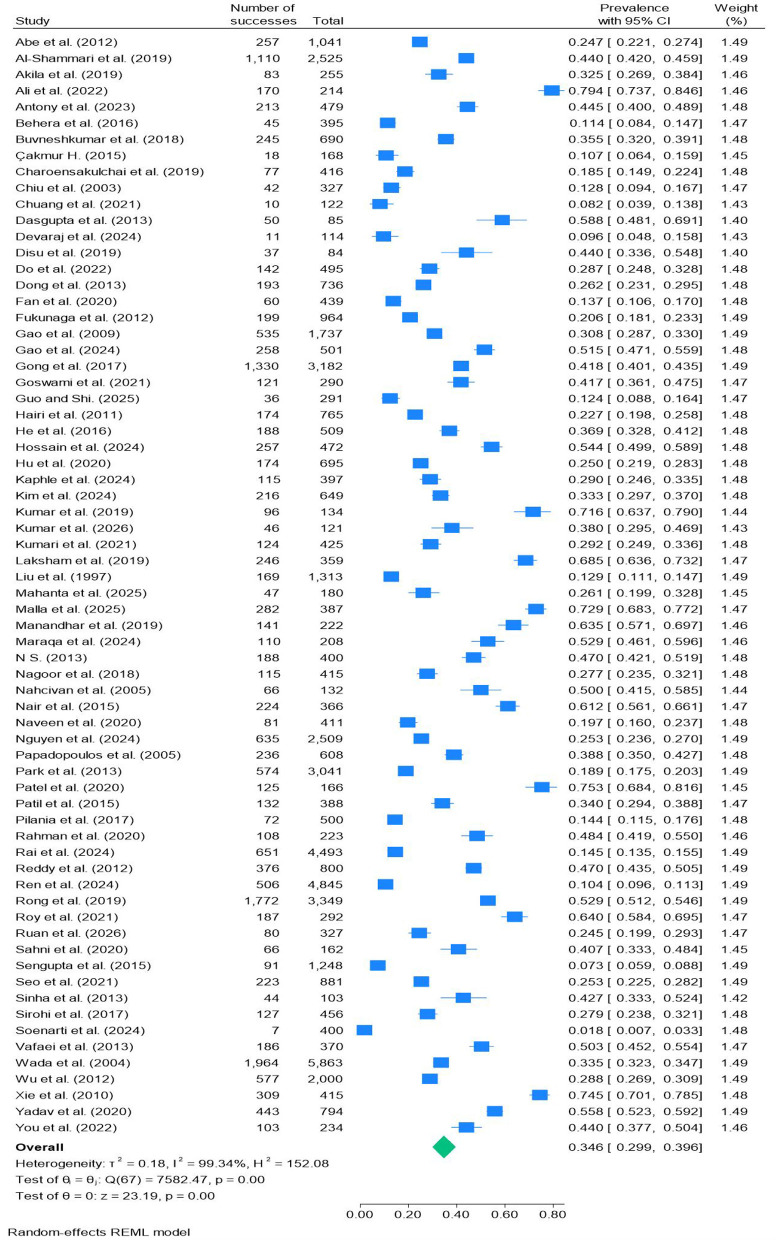
Forest plot of pooled prevalence of depression among rural older adults in Asia.

Due to the substantial heterogeneity (I^2^ = 99.3%), a random-effects model was applied, yielding a pooled prevalence of depression among rural older adults in Asia of 34.6% (95% CI: 29.9–39.6%, 95% PI: 3.9–75.9%) (Figure 2).

### 3.4 Subgroup analysis and meta-regression analysis

The results of subgroup analyses are detailed in Table 2. The estimated prevalence of depression among rural older adults in Asia differed significantly by economic status (*P* = 0.010) and geographic region (*P* < 0.001). Specifically, the pooled prevalence was 28.3% (95% CI: 22.0–35.0%) in high-income countries, 27.4% (95% CI: 20.2–35.3%) in upper-middle-income countries, and 40.9% (95% CI: 34.1–47.8%) in low-middle-income countries (Supplementary Figure S1). When analyzed by geographic region (Supplementary Figure S2), South Asia had the highest pooled prevalence (41.4%, 95% CI: 34.2–48.8%), followed by West Asia (40.3%, 95% CI: 26.9–54.3%) and East Asia (27.8%, 95% CI: 21.5–34.6%). Southeast Asia showed the lowest prevalence (17.7%, 95% CI: 7.8–30.5%). Subgroup analysis by screening tool (Supplementary Figure S3) revealed a pooled prevalence of 35.4% (95% CI: 29.5–41.5%) for studies using the GDS-15 and 32.6% (95% CI: 24.9–40.8%) for those using the GDS-30, with no significant difference between these two tools (*P* = 0.580).

**Table 2:** The results of subgroup analyses.

Subgroups	Number of studies	Prevalence (95% CI)	Heterogeneity		P values across subgroups
			*I* ^2^	*P* values	
Economic status					0.010
High income	6	28.3% (22.0–35.0%)	98.6%	*P* < 0.001	
Upper–middle income	26	27.4% (20.2–35.3%)	98.9%	*P* < 0.001	
Low–middle income	36	40.9% (34.1–47.8%)	99.4%	*P* < 0.001	
Geographic region					< 0.001
East Asia	23	27.8% (21.5–34.6%)	99.4%	*P* < 0.001	
South Asia	34	41.4% (34.2–48.8%)	99.9%	*P* < 0.001	
Southeast Asia	5	17.7% (7.8–30.5%)	98.8%	*P* < 0.001	
West Asia	6	40.3% (26.9–54.3%)	98.2%	*P* < 0.001	
Screening tool					0.580
GDS−15	49	35.4% (29.5–41.5%)	99.4%	*P* < 0.001	
GDS−30	19	32.6% (24.9–40.8%)	99.2%	*P* < 0.001	

As shown in Table 3, univariate meta-regression analysis revealed that mean age, proportion of married, economic status, and geographic region were significantly associated with the pooled prevalence of depression (all *P* < 0.05), while no significant associations were observed for sample size, proportion of female participants, or screening tool (all *P* > 0.05).

**Table 3:** Meta–regression results of of the pooled prevalence of depression among rural older adults in Asia.

Variables	Number of studies	Coefficient	Standard error	95%CI	*t* values	*P* values	*R*^2^ (%)
Publication year	68	0.0035	0.0085	(-0.0132, 0.0203)	0.41	0.680	0
Sample size	68	−7.21 × 10^−^5	4.36 × 10^−^5	(-0.0002, 0.0000)	−1.66	0.098	2.57
Mean age	33	−0.0598	0.0242	(-0.1075,−0.0123)	−2.46	0.014	6.54
Proportion of married	40	−0.0097	0.048	(-0.0192,−0.0002)	−2.01	0.044	3.32
Proportion of females	55	−0.0033	0.0053	(-0.0138, 0.0072)	−0.62	0.535	0
Sampling strategy (Ref: Probability sampling)							0
Non–probability sampling	4	0.1218	0.2000	(-0.2702, 0.5139)	0.61	0.542	
Economic status (Ref: High income)							8.01
Upper–middle income	26	−0.0180	0.1755	(-0.3275, 0.7324)	0.21	0.832	
Low–middle income	36	0.2657	0.1685	(0.0177, 0.7324)	2.06	0.040	
Geographic region (Ref: East Asia)							12.49
South Asia	34	0.2863	0.1081	(0.0744, 0.4982)	2.65	0.008	
Southeast Asia	5	−0.2417	0.1970	(-0.6279, 0.1445)	−1.23	0.220	
West Asia	6	0.2631	0.1840	(-0.0974, 0.6238)	1.43	0.153	
Screening tool (Ref: GDS−15)							0
GDS−30	19	−0.0593	0.1182	(-0.2909, 0.1724)	−0.50	0.616	
Risk of bias (Ref: Low)							0
Moderate	16	0.1288	0.1284	(-0.1229, 0.3805)	1.00	0.316	

### 3.5 Publication bias and sensitivity analysis

**Figure 3:**
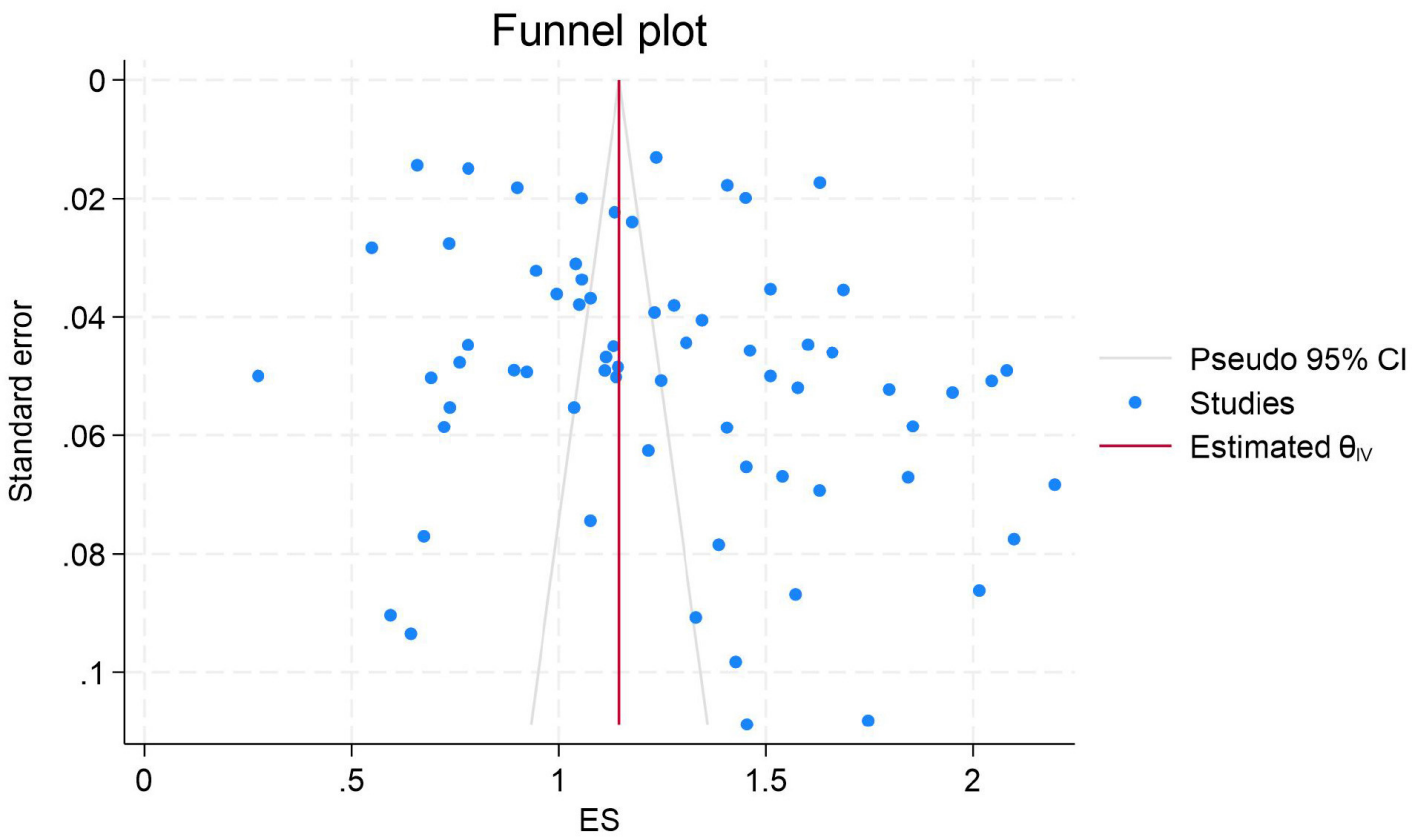
Funnel plot.

Funnel plot inspection (Figure 3) and Egger's regression test indicated significant publication bias (*Z* = 2.09, *P* = 0.037, Supplementary Figure S4). However, after further adjustment using the trim-and-fill method, no imputed studies were added, and the pooled effect size remained unchanged (Supplementary). Moreover, a modified Egger's test with the inclusion of sample size as a moderator revealed no evidence of small-study effects (*Z* = 1.27, *P* = 0.204, Supplementary Figure S6). A series of sensitivity analyses (Supplementary Figures S6–S8), including leave-one-out analysis (pooled prevalence ranging from 34.0% to 35.3%), exclusion of studies with moderate risk of bias, and exclusion of studies with missing covariate data (mean age, female proportion, and married proportion), confirmed the robustness of the pooled estimates.

## 4 Discussion

This meta-analysis aimed to determine the prevalence of depression among rural older adults in Asia. The pooled analysis of the 68 included studies demonstrated that the overall prevalence of depression was 34.6% among 58,577 rural older adults in Asia, which is higher than the global estimate reported in the meta-analyses of the general elderly population (18, 19). However, given the substantial heterogeneity observed across studies, as evidenced by the high I^2^ statistic and wide confidence and prediction intervals, caution should be warranted when applying these findings in clinical practice. The considerable burden of depression among rural older adults in Asia could be attributed to a variety of physiological and psychosocial factors. First, older adults commonly suffer from chronic conditions including hypertension, diabetes, and respiratory diseases, which may lead to persistent physical discomfort and functional impairment, both well-established risk factors for depression (14). In addition, poor nutritional status is prevalent in this population, and micronutrient deficiencies may affect the synthesis and metabolism of neurotransmitters, thereby increasing the risk of depression (76). Second, financial hardship from low income, poor pensions, and high medical costs increases depression risk by causing anxiety and helplessness (43). Meanwhile, weak social support and loneliness resulting from adult children's out-migration, widowhood, role loss, and limited community interactions further predispose this population to depression (37). Notably, limited access to mental health services may contribute to the high burden of depression among rural older adults (43, 68). Shortages of professional psychiatrists and inadequate capacity of primary healthcare workers to identify and manage depression often result in substantial underdiagnosis and untreated cases (22, 65). These findings highlight an urgent need to integrate routine depression screening into primary care and to train community health workers in evidence-based psychosocial interventions.

Our study revealed that mean age and proportion of married participants were significantly negatively associated with the pooled prevalence of depression among rural older adults in Asia. Specifically, older age and a higher proportion of married individuals were associated with a lower pooled prevalence, which is consistent with previous findings (36, 41). With increasing age, older adults gradually lower their life expectations and accept age-related functional decline as normative. They focus more on positive emotional experiences in the present rather than future losses, which may reduce the risk of depression (68). Moreover, in many Asian countries, the oldest-old adults tend to receive greater family care or government support, which may buffer depression risk (42). The protective effect of marriage against depression can be explained by several mechanisms. Spouses serve as the primary emotional attachment figures for older adults. In rural settings with limited population density and social interactions, marriage provides sustained companionship, intimacy, and opportunities for confiding, thereby reducing loneliness, a critical predictor of depression (35). Furthermore, married older adults face less financial stress than their widowed or living alone counterparts, a difference that is especially significant in rural settings where inadequate pension coverage makes the spouse a vital economic buffer (22). From a policy perspective, routine depression screening using age-specific instruments should be implemented in rural primary care, particularly for younger-old adults and those without spousal support who may be at high risk of depression.

The results of subgroup analyses and meta-regression demonstrated that economic status and geographic region served as significant moderators of the pooled prevalence of depression among rural older adults in Asia. In particular, a significantly higher prevalence was observed in low- and middle-income countries relative to high-income countries, as well as in South Asia relative to East Asia. First, low- and middle-income countries have substantially lower healthcare expenditure and mental health resource allocation, resulting in limited access to diagnostic and treatment services for depression (18). Second, economic hardship in low- and middle-income countries manifests as food insecurity, inadequate housing, and inability to afford medical care, all of which are well-documented stressors that increase depression vulnerability (68). Finally, lower levels of education and health literacy in low- and middle-income countries may lead to underrecognition of depressive symptoms, delaying appropriate intervention (32). The substantially higher depression prevalence in South Asia compared with East Asia may be explained by several region-specific factors, including lower per capita income and higher poverty rates among older adults, less developed mental health infrastructure, greater reliance on informal family care, stronger stigma associated with mental illness (49). These findings highlight the urgent need for increased mental health investment and poverty alleviation programs in low- and middle-income countries to address economic hardship and improve access to depression care.

## 5 Limitations

Several limitations should be considered. First, publication bias was observed in this study, it may be attributable to the exclusion of non-English articles and gray literature. However, after adjusting for sample size, the Egger's test was no longer significant. Furthermore, the trim-and-fill analysis did not impute any missing studies, and the pooled effect size remained unchanged, indicating that the observed bias did not materially affect the overall prevalence estimate. Second, the majority of the included studies were conducted in upper-middle-income and low-middle-income countries as well as in East and South Asia, with more than half originating from India and China. This concentration limits the generalizability of our findings to other Asian countries, as prevalence estimates may vary substantially across different cultural, economic, and healthcare contexts. Caution is warranted when extrapolating our results to under-represented Asian regions, where primary data remain scarce. Third, the finding that increasing mean age was associated with lower depression prevalence should be interpreted with caution. This analysis was based on study-level meta-regression, which examines associations across studies rather than within individuals. Therefore, this ecological finding cannot be interpreted as a protective effect of older age at the individual level, and it is susceptible to ecological fallacy. Fourth, consistent with other meta-analyses of prevalence, the pooled estimates demonstrated substantial heterogeneity and a broad prediction interval, which were only partly moderated by mean age, proportion of married participants, economic status, and geographic region in univariable meta-regression. Multivariable meta-regression was not performed due to incomplete reporting of these covariates, which limited our ability to assess their independent effects. Therefore, caution is warranted when interpreting these findings in clinical practice. Finally, all included studies used self-report measures. Despite their strong specificity and widespread use in geriatric depression research, these tools are prone to inherent biases (recall bias and social desirability bias), which may lead to either overestimation or underestimation of the true prevalence of depression.

## 6 Conclusion

Our findings reveal that more than one-third of rural older adults in Asia are affected by depression. However, the pooled prevalence estimate was associated with considerable statistical heterogeneity and a broad prediction interval, which were only partially explained by covariates including mean age, proportion of married participants, economic status, and geographic region. Therefore, this estimate should be applied with caution in clinical settings. Furthermore, given the high burden of depression in this vulnerable population, healthcare professionals should prioritize routine screening and implement targeted interventions to alleviate their depression.

## Data Availability

The original contributions presented in the study are included in the article/Supplementary material, further inquiries can be directed to the corresponding author.
