# Supplementary material for: Prevalence of depression among rural older adults in Asia: a meta-analysis of observational studies
Source: Front Public Health. 2026 Jul 8;14:1902088. doi: 10.3389/fpubh.2026.1902088 (PMC13388934; doi:10.3389/fpubh.2026.1902088)
Supplement: Supplementary file 1 [file Data_Sheet_1.docx]

**Supplemental Materials**

Table S1. The search strategies.

| Databases | Step | Search Strategies |
| --- | --- | --- |
| PubMed | #1 | "rural population"[Mesh Terms] Sort by: Best match |
|  | #2 | "rural"[Title/Abstract] OR "rural area"[Title/Abstract] OR "countryside"[Title/Abstract] OR "village"[Title/Abstract] Sort by: Best match |
|  | #3 | "aged"[Mesh Terms] Sort by: Best match |
|  | #4 | "geriatrics"[Mesh Terms] Sort by: Best match |
|  | #5 | "elderly"[Title/Abstract] OR "geriatric"[Title/Abstract] OR "older adult"[Title/Abstract] OR "older people"[Title/Abstract] Sort by: Best match |
|  | #6 | "depression"[Mesh Terms] Sort by: Best match |
|  | #7 | "depressive disorder"[Mesh Terms] Sort by: Best match |
|  | #8 | "depression"[Title/Abstract] OR "depressive symptom"[Title/Abstract] OR "depressive disorder"[Title/Abstract] Sort by: Best match |
|  | #9 | "Geriatric Depression Scale"[Title/Abstract] OR "GDS"[Title/Abstract] OR "GDS-15"[Title/Abstract] OR "GDS-30"[Title/Abstract] Best match |
|  | #10 | "prevalence"[Mesh Terms] Sort by: Best match |
|  | #11 | "incidence"[Mesh Terms] Sort by: Best match |
|  | #12 | "e[pidemiology](https://www.ncbi.nlm.nih.gov/mesh/68004813)"[Mesh Terms] Sort by: Best match |
|  | #13 | "prevalence"[Title/Abstract] OR "incidence"[Title/Abstract] OR "e[pidemiology](https://www.ncbi.nlm.nih.gov/mesh/68004813)"[Title/Abstract] Sort by: Best match |
|  | #14 | #1 OR #2 Sort by: Best match |
|  | #15 | #3 OR #4 OR #5 Sort by: Best match |
|  | #16 | #6 OR #7 OR #8 Sort by: Best match |
|  | #17 | #10 OR #11 OR #12 OR #13 Sort by: Best match |
|  | #18 | #9 AND #14 AND #15 AND #16 AND #17 Sort by: Best match |
| Web of Science | #1 | **TS=("rural population" OR "rural" OR "rural area" OR "countryside" OR "village")** |
|  | #2 | **TS=("aged" OR "geriatrics" OR "elderly" OR "geriatric" OR "older adult" OR "older people")** |
|  | #3 | **TS=("depression" OR "depressive disorder" OR "depressive symptom")** |
|  | #4 | **TS=("Geriatric Depression Scale" OR "GDS" OR "GDS-15" OR "GDS-30")** |
|  | #5 | **TS=("prevalence" OR "incidence" OR "epidemiology")** |
|  | #6 | #1 AND #2 AND #3 AND #4 AND #5 |
| Scopus | **#1** | **TITLE-ABS-KEY ("rural population" OR "rural" OR "rural area" OR "countryside" OR "village")** |
|  | #2 | **TITLE-ABS-KEY ("aged" OR "geriatrics" OR "elderly" OR "geriatric" OR "older adult" OR "older people")** |
|  | #3 | **TITLE-ABS-KEY ("depression" OR "depressive disorder" OR "depressive symptom")** |
|  | #4 | **TITLE-ABS-KEY ("Geriatric Depression Scale" OR "GDS" OR "GDS-15" OR "GDS-30")** |
|  | #5 | TITLE-ABS-KEY ("prevalence" OR "incidence" OR "e[pidemiology](https://www.ncbi.nlm.nih.gov/mesh/68004813)") |
|  | #6 | #1 AND #2 AND #3 AND #4 AND #5 |
| Embase | **#1** | **'rural population':ti,ab,kw OR 'rural':ti,ab,kw OR 'rural area':ti,ab,kw OR 'countryside':ti,ab,kw OR 'village':ti,ab,kw** |
|  | **#2** | **'aged':ti,ab,kw OR 'geriatrics':ti,ab,kw OR 'elderly':ti,ab,kw OR 'geriatric':ti,ab,kw OR 'older adult':ti,ab,kw OR 'older people':ti,ab,kw** |
|  | **#3** | **'depression':ti,ab,kw OR 'depressive disorder':ti,ab,kw OR 'depressive symptom':ti,ab,kw** |
|  | **#4** | **'Geriatric Depression Scale':ti,ab,kw OR 'GDS':ti,ab,kw OR 'GDS-15':ti,ab,kw OR 'GDS-30':ti,ab,kw** |
|  | **#5** | 'prevalence**':ti,ab,kw OR 'incidence':ti,ab,kw OR '**e[pidemiology](https://www.ncbi.nlm.nih.gov/mesh/68004813)**':ti,ab,kw** |
|  | **#6** | #1 AND #2 AND #3 AND **#4** AND **#5** |
| Cochrane Library | #1 | (rural population):ti,ab,kw OR (rural):ti,ab,kw OR (rural area):ti,ab,kw OR (countryside):ti,ab,kw OR (village):ti,ab,kw |
|  | #2 | MeSH descriptor: [Aged] explode all trees |
|  | #3 | **MeSH descriptor: [Geriatrics] explode all trees** |
|  | #4 | **(aged):ti,ab,kw OR (geriatrics):ti,ab,kw OR (elderly):ti,ab,kw OR (geriatric):ti,ab,kw OR (older adult):ti,ab,kw OR (older people):ti,ab,kw** |
|  | #5 | **MeSH descriptor: [Depression] explode all trees** |
|  | #6 | **MeSH descriptor: [Depressive Disorder] explode all trees** |
|  | #7 | **(depression):ti,ab,kw OR (depressive disorder):ti,ab,kw OR (depressive symptom):ti,ab,kw** |
|  | #8 | **(Geriatric Depression Scale):ti,ab,kw OR (GDS):ti,ab,kw OR (GDS-15):ti,ab,kw OR (GDS-30):ti,ab,kw** |
|  | #9 | **(prevalence):ti,ab,kw OR (incidence):ti,ab,kw OR (e[pidemiology](https://www.ncbi.nlm.nih.gov/mesh/68004813)):ti,ab,kw** |
|  | #10 | #2 OR #3 OR #4 |
|  | #11 | #5 OR #6 OR #7 |
|  | #12 | #1 AND #8 AND #9 AND #10 AND #11 |
| CINAHL | S1 | **TI ( "rural population" OR "rural" OR "rural area" OR "countryside" OR "village" ) OR AB ( "rural population" OR "rural" OR "rural area" OR "countryside" OR "village" ) OR SU ( "rural population" OR "rural" OR "rural area" OR "countryside" OR "village" )** |
|  | S2 | **TI ("aged" OR "geriatrics" OR "elderly" OR "geriatric" OR "older adult" OR "older people") OR AB ("aged" OR "geriatrics" OR "elderly" OR "geriatric" OR "older adult" OR "older people") OR SU ("aged" OR "geriatrics" OR "elderly" OR "geriatric" OR "older adult" OR "older people")** |
|  | S3 | **TI ( "depression" OR "depressive disorder" OR "depressive symptom") OR AB ("depression" OR "depressive disorder" OR "depressive symptom") OR SU ("depression" OR "depressive disorder" OR "depressive symptom")** |
|  | S4 | **TI ("Geriatric Depression Scale" OR "GDS" OR "GDS-15" OR "GDS-30") OR AB ("Geriatric Depression Scale" OR "GDS" OR "GDS-15" OR "GDS-30") OR SU ("Geriatric Depression Scale" OR "GDS" OR "GDS-15" OR "GDS-30")** |
|  | S5 | **TI ("prevalence" OR "incidence" OR "epidemiology") OR AB ("prevalence" OR "incidence" OR "epidemiology") OR SU ("prevalence" OR "incidence" OR "epidemiology")** |
|  | S6 | S1 AND S2 AND S3 AND S4 AND S5 |
| PsycINFO | S1 | **TI ( "rural population" OR "rural" OR "rural area" OR "countryside" OR "village" ) OR AB ( "rural population" OR "rural" OR "rural area" OR "countryside" OR "village" ) OR SU ( "rural population" OR "rural" OR "rural area" OR "countryside" OR "village" )** |
|  | S2 | **TI ("aged" OR "geriatrics" OR "elderly" OR "geriatric" OR "older adult" OR "older people") OR AB ("aged" OR "geriatrics" OR "elderly" OR "geriatric" OR "older adult" OR "older people") OR SU ("aged" OR "geriatrics" OR "elderly" OR "geriatric" OR "older adult" OR "older people")** |
|  | S3 | **TI ( "depression" OR "depressive disorder" OR "depressive symptom") OR AB ("depression" OR "depressive disorder" OR "depressive symptom") OR SU ("depression" OR "depressive disorder" OR "depressive symptom")** |
|  | S4 | **TI ("Geriatric Depression Scale" OR "GDS" OR "GDS-15" OR "GDS-30") OR AB ("Geriatric Depression Scale" OR "GDS" OR "GDS-15" OR "GDS-30") OR SU ("Geriatric Depression Scale" OR "GDS" OR "GDS-15" OR "GDS-30")** |
|  | S5 | **TI ("prevalence" OR "incidence" OR "epidemiology") OR AB ("prevalence" OR "incidence" OR "epidemiology") OR SU ("prevalence" OR "incidence" OR "epidemiology")** |
|  | S6 | S1 AND S2 AND S3 AND S4 AND S5 |

Table S2. Quality assessment results of included studies.

| Study | Q1 | Q2 | Q3 | Q4 | Q5 | Q6 | Q7 | Q8 | Q9 | Overall |
| --- | --- | --- | --- | --- | --- | --- | --- | --- | --- | --- |
| Abe et al. (2012) | Y | U | Y | Y | Y | Y | Y | Y | Y | L |
| Al-Shammari et al. (2019) | Y | Y | Y | Y | Y | Y | Y | Y | U | L |
| Akila et al. (2019) | Y | Y | Y | Y | Y | Y | Y | U | Y | L |
| Ali et al. (2022) | N | N | Y | Y | Y | Y | Y | U | U | M |
| Antony et al. (2023) | Y | Y | Y | Y | Y | Y | Y | Y | Y | L |
| Behera et al. (2016) | Y | Y | Y | Y | Y | Y | Y | Y | Y | L |
| Buvneshkumar et al. (2018) | Y | Y | Y | Y | Y | Y | Y | Y | U | L |
| Çakmur H. (2015) | Y | Y | Y | Y | Y | Y | Y | Y | U | L |
| Charoensakulchai et al. (2019) | Y | N | Y | Y | Y | Y | Y | Y | Y | L |
| Chiu et al. (2003) | Y | Y | Y | Y | Y | Y | Y | Y | Y | L |
| Chuang et al. (2021) | Y | N | Y | Y | Y | Y | Y | Y | U | L |
| Dasgupta et al. (2013) | Y | Y | Y | Y | Y | Y | Y | Y | U | L |
| Devaraj et al. (2024) | Y | Y | Y | Y | Y | Y | Y | Y | Y | L |
| Disu et al. (2019) | N | N | U | Y | Y | Y | Y | Y | U | M |
| Do et al. (2022) | Y | Y | Y | Y | Y | Y | Y | Y | U | L |
| Dong et al. (2013) | U | Y | U | Y | Y | Y | Y | Y | U | M |
| Fan et al. (2020) | Y | Y | U | Y | Y | Y | Y | Y | U | L |
| Fukunaga et al. (2012) | Y | N | U | Y | Y | Y | Y | Y | U | L |
| Gao et al. (2009) | Y | U | Y | Y | Y | Y | Y | Y | U | L |
| Gao et al. (2024) | Y | Y | U | Y | Y | Y | Y | Y | Y | L |
| Gong et al. (2017) | Y | U | U | Y | Y | Y | Y | Y | U | M |
| Goswami et al. (2021) | Y | U | Y | Y | Y | Y | Y | Y | U | L |
| Guo and Shi. (2025) | Y | Y | Y | Y | Y | Y | Y | Y | U | M |
| Hairi et al. (2011) | Y | Y | Y | Y | Y | Y | Y | Y | Y | L |
| Hossain et al. (2024) | Y | Y | Y | Y | Y | Y | Y | Y | U | L |
| He et al. (2016) | Y | U | U | Y | Y | Y | Y | Y | Y | L |
| Hu et al. (2020) | Y | U | U | Y | Y | Y | Y | Y | U | M |
| Kaphle et al. (2024) | N | N | Y | Y | Y | Y | Y | Y | U | M |
| Kim et al. (2024) | Y | Y | Y | Y | Y | Y | Y | Y | U | L |
| Kumar et al. (2019) | Y | Y | Y | Y | Y | Y | Y | Y | Y | L |
| Kumar et al. (2026) | Y | Y | Y | Y | Y | Y | Y | Y | Y | L |
| Kumari et al. (2021) | Y | Y | Y | Y | Y | Y | Y | Y | Y | L |
| Laksham et al. (2019) | Y | Y | Y | Y | Y | Y | Y | Y | Y | L |
| Liu et al. (1997) | Y | Y | Y | Y | Y | Y | Y | Y | N | L |
| Mahanta et al. (2025) | Y | Y | Y | Y | Y | Y | Y | Y | U | L |
| Malla et al. (2025) | N | N | Y | Y | Y | Y | Y | Y | U | M |
| Manandhar et al. (2019) | Y | Y | Y | Y | Y | Y | Y | Y | Y | L |
| Maraqa et al. (2024) | U | N | Y | Y | Y | Y | Y | Y | U | M |
| N S. (2013) | Y | Y | Y | Y | Y | Y | Y | Y | U | L |
| Nagoor et al. (2018) | U | U | Y | Y | Y | Y | Y | Y | U | M |
| Nahcivan et al. (2005) | Y | Y | N | Y | Y | Y | Y | Y | Y | L |
| Nair et al. (2015) | Y | Y | Y | Y | Y | Y | Y | Y | Y | L |
| Naveen et al. (2020) | Y | Y | Y | Y | Y | Y | Y | Y | U | L |
| Nguyen et al. (2024) | Y | Y | Y | Y | Y | Y | Y | Y | Y | L |
| Papadopoulos et al. (2005) | Y | Y | Y | Y | Y | Y | Y | Y | U | L |
| Park et al. (2013) | Y | Y | Y | Y | Y | Y | Y | Y | Y | L |
| Patel et al. (2020) | Y | Y | Y | Y | Y | Y | Y | Y | U | L |
| Patil et al. (2015) | Y | Y | Y | Y | Y | Y | Y | Y | U | L |
| Pilania et al. (2017) | Y | Y | Y | Y | Y | Y | Y | Y | Y | L |
| Rahman et al. (2020) | U | N | Y | Y | Y | Y | Y | Y | U | L |
| Rai et al. (2024) | Y | Y | U | Y | Y | Y | Y | Y | U | L |
| Reddy et al. (2012) | Y | Y | U | Y | Y | Y | Y | Y | U | L |
| Ren et al. (2024) | Y | Y | Y | Y | Y | Y | Y | Y | Y | L |
| Rong et al. (2019) | Y | Y | Y | Y | Y | Y | Y | Y | U | L |
| Roy et al. (2021) | Y | N | U | Y | Y | Y | Y | Y | N | M |
| Ruan et al. (2026) | Y | Y | Y | Y | Y | Y | Y | Y | U | L |
| Sahni et al. (2020) | Y | Y | Y | Y | Y | Y | Y | Y | U | L |
| Sengupta et al. (2015) | Y | Y | Y | Y | Y | Y | Y | Y | Y | L |
| Seo et al. (2021) | Y | N | U | Y | Y | Y | Y | Y | U | M |
| Sinha et al. (2013) | Y | Y | Y | Y | Y | Y | Y | Y | Y | L |
| Sirohi et al. (2017) | Y | Y | Y | Y | Y | Y | Y | Y | Y | L |
| Soenarti et al. (2024) | Y | U | Y | Y | U | Y | Y | Y | U | M |
| Vafaei et al. (2013) | Y | Y | Y | Y | Y | Y | Y | Y | Y | L |
| Wada et al. (2004) | Y | Y | Y | Y | Y | Y | Y | Y | Y | L |
| Wu et al. (2012) | Y | Y | Y | Y | Y | Y | Y | Y | U | L |
| Xie et al. (2010) | Y | U | U | Y | Y | Y | Y | Y | U | M |
| Yadav et al. (2020) | Y | Y | Y | Y | Y | Y | Y | Y | U | L |
| You et al. (2022) | Y | Y | Y | Y | Y | Y | Y | Y | U | L |

Notes: Y: Yes, N: No, U: Unclear. L: Low risk, M: Moderate risk, H: High risk.

Q1: Was the sample frame appropriate to address the target population?

Q2: Were study participants sampled in an appropriate way?

Q3: Was the sample size adequate?

Q4: Were the study subjects and the setting described in detail?

Q5: Was the data analysis conducted with sufficient coverage of the identified sample?

Q6: Were valid methods used for the identification of the condition?

Q7: Was the condition measured in a standard, reliable way for all participants?

Q8: Was there appropriate statistical analysis?

Q9: Was the response rate adequate, and if not, was the low response rate managed appropriately?

Each study was categorized by risk of bias as follows: high risk if ≤ 49% of items are responded to with "yes;" moderate risk if 50%-69% of items are responded to with "yes; "and low risk if ≥ 70% of items are responded to with "yes."

Figure S1. The pooled prevalence of depression among rural older adults in Asia based on economic status.

Figure S2. The pooled prevalence of depression among rural older adults in Asia based on geographic region.

Figure S3. The pooled prevalence of depression among rural older adults in Asia based on screening tool.


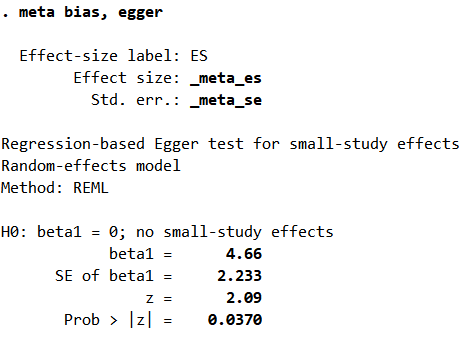


Figure S4. Egger's test results.


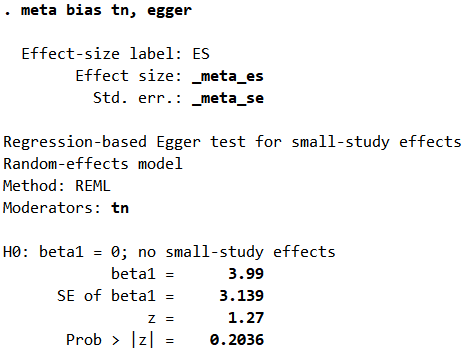


Figure S5. Egger's test with moderator sample size.


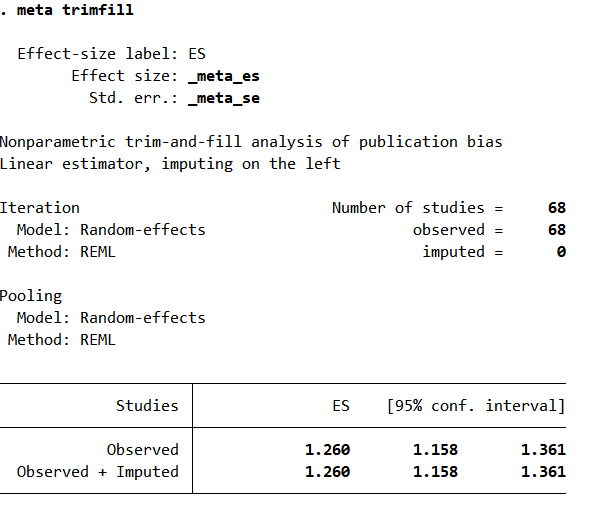


Figure S6. Trim-and-fill analysis results.

Figure S7. Leave-one-out sensitivity analysis

Figure S8. Sensitivity analysis restricted to studies with complete data on mean age.

Figure S9. Sensitivity analysis restricted to studies with complete data on proportion of married participants.

Figure S10. Sensitivity analysis restricted to studies with complete data on proportion of females.

Figure S11. Sensitivity analysis restricted to low risk of bias studies.
